# Factors that influence acute malnutrition detection and treatment by community health promoters in Samburu and Turkana counties, Kenya: A mixed methods study

**DOI:** 10.1371/journal.pgph.0005689

**Published:** 2026-01-21

**Authors:** Monica Nthamba Ng’ang’a, Faith Thuita, Moses Ngari, Albert Webale, Teresia Macharia, Simon Eris, Geoffrey Wafula, Lominito Lomoru, Rita Mukoma, Hassan Mohamed, Valerie L. Flax

**Affiliations:** 1 RTI International, Nairobi, Kenya; 2 Department of Public and Global Health, University of Nairobi, Nairobi, Kenya; 3 Clinical Research Department, KEMRI/Wellcome Trust Research Programme, Kilifi, Kenya; 4 Department of Public Health, School of Health and Human Sciences, Pwani University, Kilifi, Kenya; 5 International Rescue Committee, Nairobi, Kenya; 6 Mercy Corps, Nairobi, Kenya; 7 UNICEF, Nairobi, Kenya; 8 Save the Children International, Nairobi, Kenya; 9 RTI International, Durham, North Carolina, United States of America; PLOS: Public Library of Science, UNITED STATES OF AMERICA

## Abstract

Global acute malnutrition (GAM) among children <5 years contributes to mortality and is a persistent problem in the drylands of Africa, including Samburu and Turkana Counties in Kenya, due to recurring droughts and other factors. In 2023, GAM prevalence was 20.3% in Samburu and 34.8% in Turkana, far exceeding the emergency threshold of 15%. Community health promoters (CHPs) are frontline members of the community health system who work with families to detect, treat, and refer acute malnutrition cases, but little is known about factors associated with these tasks. We conducted a mixed-method study with a convergent parallel design as part of the Nawiri program in Samburu and Turkana Counties to better understand how training, supervision, self-efficacy, social and peer support, supplies and equipment, stipends, and motivation affect whether CHPs carry out GAM detection and treatment. Outcome data on GAM detection and treatment were compiled from CHPs’ monthly reports. Survey data on factors that influence GAM detection and treatment were collected from all CHPs in selected community health units on the same days as monthly meetings. The quantitative data were analyzed using structural equation modeling. Focus group discussions with CHPs to gather information that complemented the survey data and key informant interviews with community health assistants to understand the challenges CHPs face were conducted and analyzed using thematic content analysis methods. A total of 490 CHPs were enrolled – more than half were women. CHP self-efficacy and work non-self-determined motivation (W-NDSM) were positively associated with GAM detection but work self-determined motivation (W-SDM) had no direct effect on GAM detection. CHP self-efficacy, W-SDM, and W-NDSM had no direct effect on GAM treatment. Qualitative findings revealed that CHPs were motivated by support received through supervision, social and peer networks, and community recognition. However, CHPs faced challenges such as irregular training sessions, insufficient supplies, and community hostility. Strengthening CHP training, supervision, and support systems is essential for improving the detection and treatment of acute malnutrition. Addressing barriers such as supply shortages and community hostility, while enhancing motivation through recognition and support, may lead to better detection and treatment of acute malnutrition in these counties.

## Introduction

Global acute malnutrition (GAM) among children <5 years contributes to morbidity and mortality [1,2]. GAM is the presence of both severe acute malnutrition (SAM) and moderate acute malnutrition (MAM) based on a weight-for-height z-score or mid-upper arm circumference (MUAC) below accepted thresholds [3]. In 2024, 42.8 million children <5 years were estimated to have GAM globally, including 11.7 million in Africa [4]. A high prevalence of GAM is persistent in Africa’s arid and semi-arid areas, including Samburu and Turkana Counties in Kenya, because of recurring and prolonged droughts, livelihood systems that are sensitive to climate shocks, weak institutions, inadequate social safety nets, and ongoing conflicts [5]. In 2023, the prevalence of GAM was 20.3% in Samburu and 34.8% in Turkana [6]. These prevalence estimates are far higher than the emergency threshold of 15% [5].

Children diagnosed with SAM need intervention either in the community using ready-to-use therapeutic food (RUTF) or at a health facility if complications arise, while those with MAM need access to a nutrient-dense diet and, in some cases, specially formulated food interventions with counseling [7]. Throughout this paper, we refer to recommended interventions for children with SAM or MAM as “treatment.” Community-based management of acute malnutrition (CMAM) and family-led mid-upper arm circumference (MUAC) are two strategies for early detection, referral, and treatment of children with acute malnutrition. CMAM is an approach aimed at treating acute malnutrition in children within their communities [8,9]. CMAM allows community health promoters (CHPs) to manage acute malnutrition at community level by providing them with training, MUAC tapes, medicines, and nutritional supplements (e.g., RUTF or specially formulated foods) [10]. Family-led MUAC involves training mothers and other family members to use a MUAC tape to monitor their children’s nutritional status every two weeks [11]. Families detecting SAM or MAM based on MUAC measurements can then seek treatment for the child from the CHP or referral to a healthcare facility.

Detecting, treating, and monitoring acute malnutrition cases early to prevent worsening of the condition and to lessen default or relapse after treatment is important in addressing GAM. The community health system (CHS), which constitutes the lowest level of the healthcare delivery system in Kenya, is a crucial bridge between community members and essential healthcare services for early detection and treatment of acute malnutrition. Following national guidelines, the CHS in each county in Kenya is structured around community health units (CHUs), each catering to a population of 5,000 people [12]. Within these CHUs, there is a mandate to have one dedicated CHP for every 25 households. CHPs are overseen by a community health assistant (CHA), with each CHA supervising 25 CHPs. The CHPs serve as the initial point of contact for community members seeking healthcare services, making them integral to the functioning of the healthcare system. When the study was conducted, there were 45 CHUs with 1,438 CHPs in Samburu and 208 CHUs with 2,268 CHPs in Turkana. At the time of the study, Turkana County provided a monthly stipend to CHPs [13], while Samburu County had recently passed a bill legislating a stipend for CHPs [14].

The Nawiri program in Samburu and Turkana Counties was designed to address the multisectoral factors that contribute to the persistently high prevalence of GAM in these counties. As part of Nawiri, we developed a conceptual model to underpin the design of programmatic activities within the CHS to motivate and support CHPs to more effectively carry out tasks related to acute malnutrition detection, referral and treatment. The analysis used empirical data to test the strength of the associations between constructs in our conceptual model and to complement findings with qualitative data that helped to explain the results.

## Methods

### Study design and setting

This study employed a convergent parallel mixed-methods design [15]. This design was selected because it involves collection of quantitative and qualitative data at the same time, separate analysis of the two types of data, and then integration to identify points of convergence or divergence that help to form a comprehensive picture of the research topic. The quantitative component of this study used monthly data reported by CHPs on their GAM screening and treatment activities and survey data collected from CHPs before and during the Nawiri program in selected CHUs in Samburu and Turkana Counties. The quantitative data were used to carry out structural equation modeling (SEM) to assess the associations of a variety of factors with CHPs’ acute malnutrition detection and treatment. The qualitative component included focus group discussions (FGDs) with CHPs to gather information that complemented the survey data and key informant interviews (KIIs) with CHAs to understand the challenges CHPs face when trying to screen for and treat acute malnutrition.

The study was conducted in Samburu and Turkana Counties, arid and semi-arid areas in northwestern Kenya. The population in these counties is spread across vast areas and the main livelihoods are pastoral, agropastoral, urban/peri-urban, and fishing (Turkana only). Access to health facilities is limited by long distances and lack of transportation, making CHPs an important source of basic health care, screening, and information. Recurrent droughts and food insecurity are persistent in both counties.

### Conceptual framework

**Fig 1:**
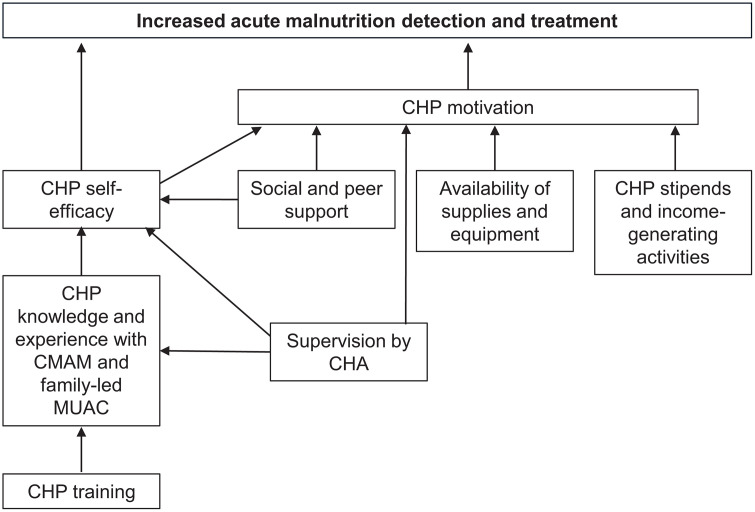
Conceptual framework of factors that influence CHPs to carry out acute malnutrition detection and treatment.

This research was guided by the conceptual framework developed for this component of the Nawiri program (**Fig 1**). The program expected that supporting the county CHS teams to strengthen the health system would increase CHP self-efficacy and motivation, which would contribute to their ability to implement Integrated Community Case Management - Community-Based Management of Acute Malnutrition (ICCM-CMAM) and family-led MUAC activities, thereby increasing acute malnutrition detection and treatment. CHP self-efficacy would be increased through improvement in CHP training and supervision, which would lead to increased CHP knowledge and experience with CMAM and family-led MUAC. Social support to CHPs from their communities, peers, and CHAs would also contribute to CHP self-efficacy. CHP motivation would be increased through increased self-efficacy to carry out CMAM and family-led MUAC activities, social and peer support, supervision, necessary supplies and equipment, monthly stipends, and income-generating activities.

### Eligibility

Individuals were eligible for the survey or FGDs if they were currently working as CHPs in the CHUs selected for the study, 18 years or older, and willing to participate in the research and provide informed consent. Individuals were eligible for KIIs if they worked as CHAs in the selected CHUs, were 18 years or older, and willing to participate in the research and provide informed consent.

### Sampling and sample size

The study was conducted in 30 CHUs in Samburu and Turkana Counties. Within each county, three CHUs were selected per subcounty based on predominant livelihood, CHU functionality, and acute malnutrition caseload. A list of the CHUs included in the study is found in **S1 Table**.

The study included all 490 CHPs in the selected CHUs (census sampling). With a sample of 490 CHPs, the study was powered to detect at least a 20% prevalence of children with GAM (based on prevalence of GAM in Samburu County, which was lower than in Turkana County), assuming precision of 5%, two-tailed alpha of 0.05, and 10% non-responses/missing outcome data.

Purposive sampling, based on engagement with the program, was used to select eligible CHPs and CHAs for FGDs and KIIs. Purposive sampling is commonly used in qualitative research to ensure that participants have experience relevant to the research questions so that they can provide in-depth insights. The distribution of the qualitative participants was as follows: 17 FGDs with CHPs (3 Samburu, 14 Turkana) and 13 KIIs with CHAs (6 Samburu, 7 Turkana). Samburu is smaller geographically and in population size than Turkana and fewer CHUs were included in Samburu than Turkana. This explains the variation in the number of participants between the counties. The number of qualitative participants was selected to achieve saturation. Saturation is the point in qualitative research at which no new information is obtained through additional data [16].

### Data collection

Surveys were conducted with CHPs on the same days as CHPs’ monthly meetings with CHAs at health facilities and were scheduled with individual CHPs before or after the meetings. Participants were enrolled from September 17 to October 3, 2021. Trained data collectors administered the survey and entered the data on tablets using SurveyCTO. Topics covered in the survey questionnaire included: CHP training, supervision, motivation, social support, self-efficacy, quality of care, supplies and equipment, and stipends and income-generating activities. The tool was administered in local languages.

The outcome data (GAM assessment and treatment) was submitted by CHPs in their monthly reports to CHAs. It was compiled and checked by Save the Children, the Nawiri implementing partner responsible for health-related components of the program.

Qualitative data were collected from CHPs through FGDs and from CHAs through KIIs. The FGDs were led by a moderator, who was assisted by a notetaker, and conducted in a rented hall, classroom, or church. The KIIs were conducted by an interviewer in private rooms, at the CHAs’ offices, or online via video/audio calls. FGDs and KIIs were digitally recorded with permission of participants. The recordings were transcribed and translated into English. The notetaker’s notes served as a backup and helped to guide the transcriptionist. Topics covered in the discussion guide included: CHP training, supervision, motivation, social support, self-efficacy, quality of care, supplies and equipment, and stipends and income-generating activities. The tools were administered in local languages. The qualitative data were collected from November 2020 to June 2022.

### Outcome variables

The main study outcomes were percentage of children detected with GAM among all children screened by CHPs and percentage of children detected with GAM who were treated, using the data reported by CHPs. GAM assessment and treatment data was gathered from CHPs for three months following the survey and the values were averaged for each CHP. GAM was defined as total prevalence of SAM and MAM among children under 5 years old.

### Latent exposure variables

We explored the pathways to the main outcomes using our *a priori* conceptual framework (**Fig 1**). The pathways were analyzed using eight estimated latent variables: CHP training, supervision by CHA, CHP knowledge and experience with CMAM and family-led MUAC, CHP self-efficacy, social and peer support, availability of supplies and equipment, CHP stipends and involvement in income-generating activities, and CHP motivation (work self-determined motivation (W-SDM) and work non-self-determined motivation (W-NDSM) [17]. The methods of computing the latent variables are summarized below and described in detail in **S2 Table**. All eight latent variables were included as continuous scores.

CHP training was calculated by summing a total of 16 training modules. The CHPs were given a score of one for each training completed or zero if the training was not completed. Supervision by CHA was calculated by summing the number of supervision activities involving five different items. Each supervision activity response was a discrete count of the number of supervisions. CHP knowledge and experience with CMAM and family-led MUAC was calculated by summing responses to seven questions about CHPs’ CMAM and family-led MUAC triage and treatment knowledge and experience. CHP self-efficacy was calculated by summing responses to eight questions. Each question had a 5-point scale, ranging from a score of one for very low level of confidence to a score of five for very high level of confidence. Social and peer support was calculated by summing responses to 11 social support and 4 peer support questions. The questions had three levels: one for never, two for sometimes, and three for always. Availability of supplies and equipment was calculated by summing yes/no responses to 34 essential supplies and equipment questions. The responses were scored as zero if absent and one if present. CHP stipends and income-generating activities was calculated by summing two yes/no questions, one asking about receipt of a stipend from the government or implementing organizations and the other about engagement in income-generating activities. CHP motivation was calculated by summing W-SDM (a sum of means of intrinsic motivation (IM), integrated regulation (INTEG) and identified regulation (IDEN)) and W-NSDM (as sum of means of introjected regulation (INTRO), external regulation (EXT) and amotivation (AMO)).

### Data analysis

Participant characteristics were described using frequencies and percentages. For continuous variables, mean (SD) and median (IQR), depending on the distribution, were reported. The percentage of children detected with GAM and treated were reported with 95% exact binomial confidence intervals.

SEM was used to investigate the pathways leading to either acute malnutrition detection or treatment based on the conceptual framework (**Fig 1**). A total of four models were constructed: two for each outcome (acute malnutrition detection and acute malnutrition treatment) one going through W-SDM and the other going through and W-NSDM. Because acute malnutrition detection and treatment were continuous scores, linear regression within SEMs was modelled and adjusted regression coefficients reported as the measure of effects. The SEM with acute malnutrition treatment as the main outcome only included records where GAM was detected. SEM goodness of fit was assessed using chi-square value (a good fit will have P ≥ 0.05), root mean square error of approximation (RMSEA, a value <0.08 indicates good fit), and standardized root mean square (SRMR, values <0.08 are acceptable) [18,19]. STATA Version 17.0 (College Station, TX, USA) was used to perform SEM and *DiagrammeR* in R statistical software (version 4.0.2) was used to visualize the SEM network diagrams. Statistical significance was evaluated using a two-tailed P-value <0.05.

Qualitative data was analyzed with Nvivo software Version 12.4.0 (Denver, CO, USA), using thematic content analysis methods [20]. Codebooks were developed for each type of participant using deductive codes based on the discussion guides and inductive codes were added as needed during the coding process. Codes were grouped into key themes and code reports were used to create summary memos for each theme.

### Ethical considerations

Ethical approval for the study was obtained from the ethics and scientific review committee of Amref Health Africa (P1005-2021). A reliance agreement was signed between Amref and Research Triangle Institute’s institutional review board. Written informed consent was obtained from all participants. Participation was voluntary, and the risks and benefits of the study were explained to all study participants. No incentives were provided to CHAs because they were employed by the counties or to CHPs who participated in the surveys because they received training and other support from the program. However, CHPs who participated in FGDs received snacks and 500 Kenyan shillings for transport.

## Results

### Participant characteristics

A total of 490 CHPs were enrolled in the study, 56% and 44% from Turkana and Samburu Counties, respectively (**Table 1**). Sixty-two percent were female. They had worked as CHPs for a median of 8 years. Nearly seventy percent had a primary education or lower, while more than 80% had an occupation in addition to working as a CHP.

**Table 1:** CHPs’ sociodemographic characteristics.

Characteristics	n (%) (N = 490)
**Age in years**	
<30	119 (24)
30–39	168 (34)
40–49	154 (31)
≥50	49 (10)
**Sex**	
Male	187 (38)
Female	303 (62)
**County**	
Turkana	276 (56)
Samburu	214 (44)
Years of work as CHP, median (IQR)	8 (4-10 )
**People living in the household**	
1–5	101 (21)
6–10	309 (63)
>10	80 (16)
**Marital status**	
Married	426 (87)
Single	64 (13)
**Education level**	
No formal education	60 (12)
Primary incomplete	159 (32)
Primary complete	125 (25)
Secondary incomplete	51 (10)
Secondary complete	57 (12)
Post-secondary/college	27 (6)
Adult education	11 (2)
**Occupation (in addition to CHP roles)**	
Livestock herding	67 (14)
Crop farming	36 (7)
Employed	15 (3)
Wage labor	64 (13)
Petty trade	135 (28)
Trader	43 (9)
Self-employed	42 (9)
Domestic work	11 (2)
Unemployed	77 (16)

All results are n (%) unless specified. CHP, community health promoter; IQR, interquartile range.

### Outcome and latent variables

The percentage of children detected with GAM among all children screened was 25% (95% CI 22–28). Among those detected with GAM, 39% (95% CI 32–46) were treated.

Scores of the latent variables used in the models are shown in **Table 2**. Details of the components that were used to create the latent variables are reported in **S3****-****S10 Tables**. The median value and IQR for each latent variable were: CHP trainings (12, IQR, 9–14); supervision visits by CHA (8, IQR, 4–12); CHP knowledge of CMAM and family-led MUAC (8, IQR, 8–9); CHP self-efficacy (35, IQR, 31–38); social and peer support (30, IQR, 36–34); availability of supplies and equipment (7, IQR, 4–10); CHP stipends and income generating activities (1, IQR, 0–1); W-SDM (13, IQR, 11 to 14); and W-NSDM (9, IQR, 8 to 11).

**Table 2:** Scores of factors that influence CHPs’ performance of acute malnutrition detection and treatment.

Characteristics	Median (IQR) (N = 490)
CHP trainings completed	12 (9‒14)
CHP supervision visits	8 (4‒12)
CHP CMAM and family-led MUAC knowledge	8 (8‒9)
CHP self-efficacy	35 (31‒38)
CHP social and peer support	30 (26‒34)
CHP availability of supplies and equipment	7 (4‒10)
CHP stipends and income generating activities	1 (0‒1)
**CHP motivation**	
Work self-determined motivation (W-SDM)	13 (11‒14)
Work nonself-determined motivation (W-NSDM)	9 (8‒11)

CHP trainings completed, sum of 16 trainings completed, the range is from zero to 16; CHP supervision visits, count of number of supervision activities (no range); CHP CMAM and family-led MUAC knowledge, sum of nine questions on knowledge of CMAM and family-led MUAC (yes/no); CHP self-efficacy, sum of eight questions about CHP self-efficacy, the range is from 8 to 40; CHP social and peer support, sum of responses from the social support and peer support, the range is from one to 37; CHP availability of supplies and equipment, sum of 34 essential supplies available, range from zero to 34; CHP stipends and income generating activities, sum of two questions on receipt of stipends and involvement in any income generating activity, range from zero to two; W-SDM (work self-determined motivation), sum of means of intrinsic motivation (IM), integrated regulation (INTEG) and identified regulation (IDEN); and W-NSDM (work nonself-determined motivation), sum of means of introjected regulation (INTRO), external regulation (EXT) and amotivation (AMO).

CHP trainings completed, sum of 16 trainings completed, the range is from zero to 16; CHP supervision visits, count of number of supervision activities (no range); CHP CMAM and family-led MUAC knowledge, sum of nine questions on knowledge of CMAM and family-led MUAC (yes/no); CHP self-efficacy, sum of eight questions about CHP self-efficacy, the range is from 8 to 40; CHP social and peer support, sum of responses from the social support and peer support, the range is from one to 37; CHP availability of supplies and equipment, sum of 34 essential supplies available, range from zero to 34; CHP stipends and income generating activities, sum of two questions on receipt of stipends and involvement in any income generating activity, range from zero to two; W-SDM (work self-determined motivation), sum of means of intrinsic motivation (IM), integrated regulation (INTEG) and identified regulation (IDEN); and W-NSDM (work nonself-determined motivation), sum of means of introjected regulation (INTRO), external regulation (EXT) and amotivation (AMO).

### SEM models

**Fig 2:**
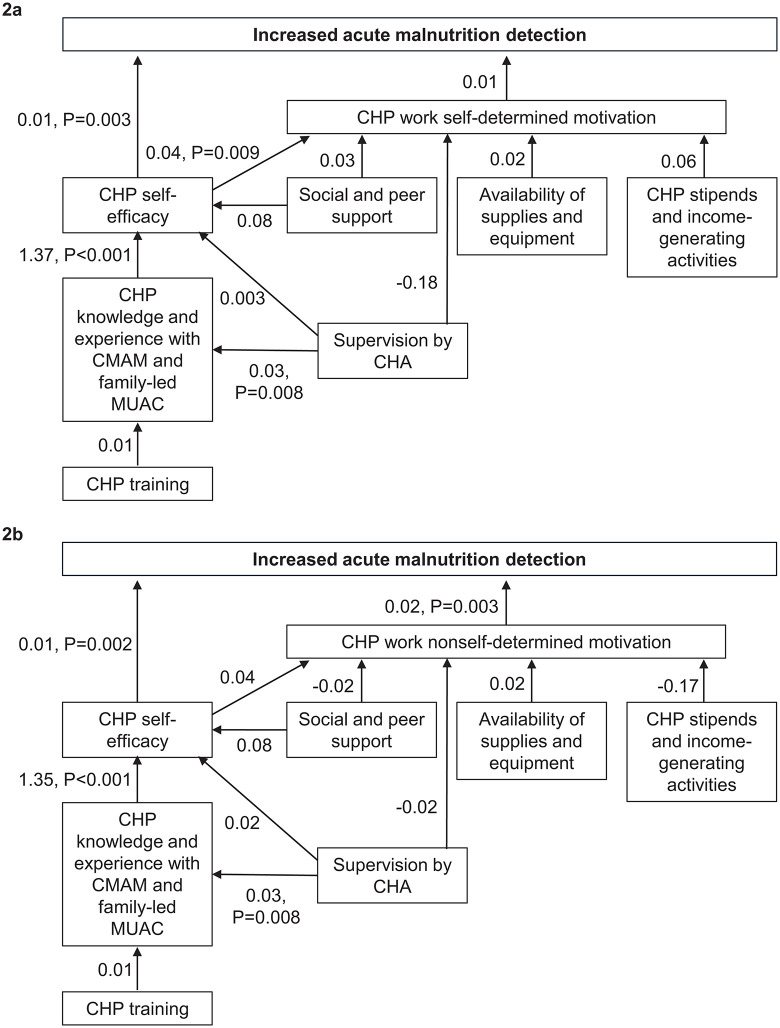
Structural equation models showing pathways to acute malnutrition detection through work self-determined motivation (2a) and work nonself-determined motivation (2b). The values on the pathways are the regression coefficients. Significant pathways (P < 0.05) are shown with both the regression coefficients and the P-value.

**Fig 2a** and **S11 Table** show the associations among the pathways to acute malnutrition detection through W-SDM. CHP self-efficacy was positively associated with acute malnutrition detection, but W-SDM was not associated. CHP knowledge of CMAM and family-led MUAC was positively associated with self-efficacy, and social and peer support had a borderline positive effect on CHP self-efficacy, while supervision by a CHA was not associated with CHP self-efficacy. Supervision by a CHA was positively associated with CMAM/MUAC knowledge and experience but CHP training was not associated. CHP self-efficacy was positively associated with W-SDM, but all other factors in the framework were not associated with W-SDM.

**Fig 2b** and **S12 Table** show the associations among the pathways to acute malnutrition detection through W-NSDM. CHP self-efficacy and W-NSDM were positively associated with acute malnutrition detection. Supervision by a CHA was positively associated with CHP CMAM/MUAC knowledge, and CMAM/MUAC knowledge was positively associated with CHP self-efficacy, but other factors were not associated with CHP self-efficacy. None of the other factors in the framework were associated with W-NSDM.

**Fig 3:**
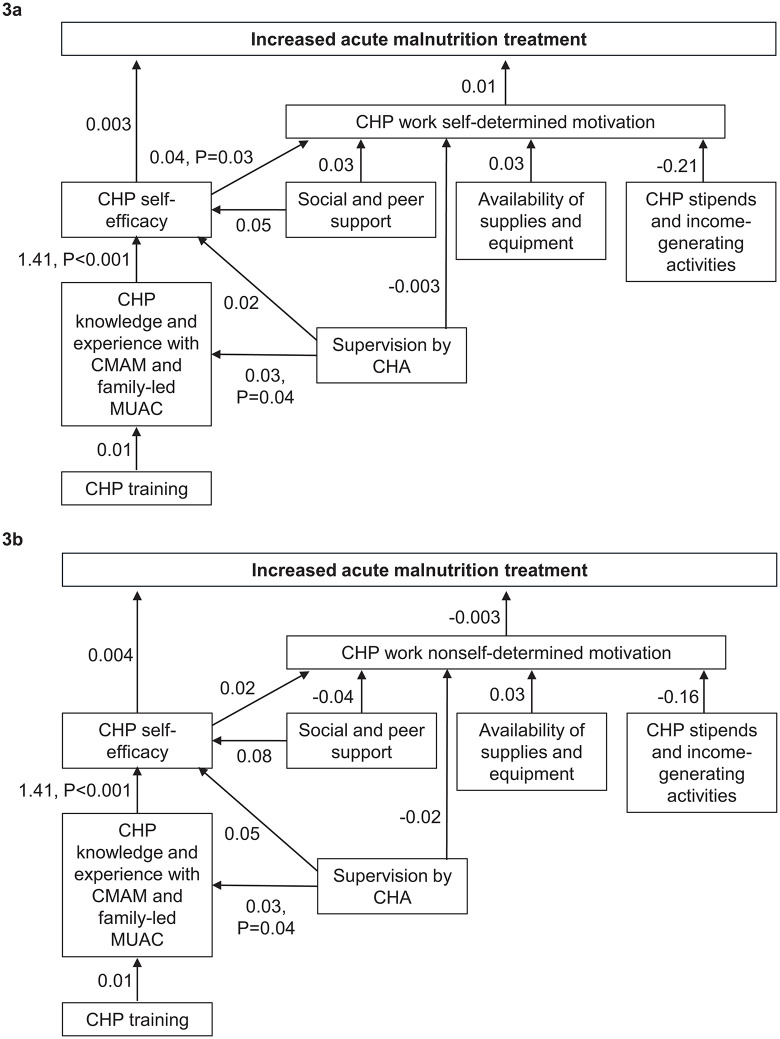
Structural equation models showing pathways to acute malnutrition treatment through work self-determined motivation (3a) and work nonself-determined motivation (3b). The values on the pathways are the regression coefficients. Significant pathways (P < 0.05) are shown with both the regression coefficients and the P-value.

**Fig 3a** and **S13 Table** show the associations among the pathways to acute malnutrition treatment through W-SDM. Neither CHP self-efficacy nor W-SDM were associated with acute malnutrition treatment. Supervision by a CHA was positively associated with CHP CMAM/MUAC knowledge and CMAM/MUAC knowledge was positively associated with CHP self-efficacy. CHP self-efficacy was positively associated with W-SDM. No other factors in the framework were significantly associated.

**Fig 3b** and **S14 Table** show the associations among the pathways to acute malnutrition treatment through W-NSDM. The pattern of associations was the same as for Fig 3a, in that CHP self-efficacy and W-NSDM were not associated with acute malnutrition treatment, supervision by a CHA was positively associated with CHP CMAM/MUAC knowledge, and CMAM/MUAC knowledge was positively associated with CHP self-efficacy. The only difference was that CHP self-efficacy was not associated with W-NSDM.

## Qualitative findings

Eighty CHPs participated in FGDs. The participants included both male and female CHPs, aged 20–65 years. Their experience as CHPs ranged from 5 months to 15 years. A total of 13 CHAs participated in KIIs.

Qualitative findings were grouped into themes related to training, supervision, social support, peer support, supplies and equipment, stipends, and experience with CMAM and family-led MUAC. Illustrative quotations for each theme are included in **Table 3**.

**Table 3:** Qualitative quotes by themes.

Theme	Illustrative quotes
**Training**	• *“After trainings, CHPs are given certificates so that when they are out, they can show that they have been trained.”* CHA, Samburu Central • *“Distance also affects* *CHP training attendance because some of the CHPs live far. Some walk and arrive late or miss completely.”* CHA, Turkana East
**Supervision**	• *“Having the CHA helps them in report writing and being closely monitored gives them the motivation to work.”* CHA, Samburu North • *“Supervision of some CHPs is difficult because they are working from outstations in distant areas like Nadopua, Natoot, Mlango, and face poor [mobile phone] networks for communication.”* CHA, Turkana North
**Social support**	• *“Sometimes during community barazas (meetings), the chief recognizes CHPs’ work and gives them a chance to give health talks to the community.”* CHP, Samburu East • *“Sometimes we get resistance from our community members because they believe we benefit ourselves [as CHPs by] using their names.”* CHP, Turkana Central
**Peer support**	• *“When I find a pregnant woman in labor, I attend to her without checking the CHU she is from [to help my fellow CHPs].”* CHP, Turkana West • *“Male CHPs are not able to teach some things to women, some topics are sensitive and only female CHPs can handle them.”* CHP, Samburu North
**Supplies and equipment**	• *“The supplies and equipment run out and might take long before we are equipped with them again.”* CHP, Samburu East • *“Most CHPs use MUAC tapes that were given to them some five years ago, so those MUAC tapes are worn out now. As a result of this, there is inaccuracy of their data.”* CHA, Turkana North
**Stipends**	• *“AMREF has been motivating CHPs by giving them Ksh. 2,000 per month.”* CHA, Samburu East • *“Delayed stipends discourage me as a CHP in conducting my activities because we also have families to cater for.”* CHP, Turkana Central
**Experience with CMAM and family-led MUAC**	• *“Some mothers fear having their children measured because they fear stigmatization in the community [if their children are found to be malnourished].”* CHA, Loima • *“Failure of parents to bring children for growth monitoring and promotion so [acute malnutrition] could be detected earlier.”* CHA, Samburu East

### Training

Most CHPs reported completing some training sessions. These sessions covered CHP induction and technical aspects of the work, and primarily emphasized maternal, infant, and young child nutrition and care. Additionally, the trainings encompassed topics such as integrated management of acute malnutrition; tracing and follow-up of acute malnutrition defaulter cases; family planning; Ministry of Health data collection tools; household registration and visits; drug and supplement administration; water, sanitation, and hygiene; community engagement activities; and the roles and responsibilities of CHPs.

CHPs generally expressed satisfaction with the induction trainings, finding them informative and adequately preparing them for their upcoming tasks. These sessions enhanced their confidence in health knowledge and allowed them to witness the impact of their work within their communities. According to most key informants, the trainings facilitated the transfer of knowledge from CHPs to community members, thereby enhancing service delivery in the community.

Participants explained that trainings contributed to CHPs’ motivation through the skills they gained, provision of training certificates, meals provided, and training allowances offered by implementing partners. Among the most cited barriers to effective CHP training were the irregularity of training sessions, the absence of regular refresher courses, varying levels of literacy among CHPs, difficulty comprehending materials due to complex language used during training, and the distance to training venues.

### Supervision

Participants emphasized the necessity of regular and dependable supervision for the success of any CHP activity. According to most key informants, when CHAs or occasionally subcounty government officials oversee CHPs, improvements are observed across various areas including patient referral, identification of acute malnutrition cases, tracing of acute malnutrition defaulters (e.g., cases detected who do not seek treatment or who do not complete treatment), community engagement activities like action days and dialogues, timely submission of reports, household visits, attendance at monthly meetings, and CHP motivation.

Regular individual and group supervisory sessions facilitate effective collaboration between CHAs and CHPs, enabling them to address challenges together. Most CHPs said they were comfortable with supervision. They also noted that supervision helps address concerns directly on-site.

Supervision by CHAs motivates CHPs as it offers guidance in areas where they may lack expertise, such as reporting. The most cited barriers to effective supervision include the distance to communities and the lack of transport for CHAs.

### Social support

Social support from various entities, including CHAs and health facility staff, served as a significant source of motivation for CHPs. Additionally, CHPs felt motivated when community members actively participated in community engagement activities led by them, adhered to the information provided by CHPs, and disseminated information within the community.

Recognition and respect from CHUs and partners through recognition messages and from community members and leaders contributed to the motivation of CHPs. Some CHPs expressed gratitude for recognition received for their efforts, such as acknowledgements by chiefs during chief barazas (community meetings). Others mentioned receiving support from their communities in various forms, including financial assistance, provision of transportation, or offering food and drinks.

The primary obstacle related to social support for CHPs was encountered when hostility arose from the community. This occurred when community members perceived CHPs as benefiting financially in their role as CHPs.

### Peer support

According to most CHPs, they mainly support one another in filling reports and screening for acute malnutrition. They also support one another by covering for absent colleagues, sharing knowledge with those who have not received training, managing patients belonging to other CHPs, reminding each other of trained topics, borrowing supplies and commodities from each other, establishing savings groups, assisting in weighing children during outreach programs, providing both social and financial assistance, exchanging ideas, tracing defaulters, and distributing MUAC tapes to community members. Nevertheless, most CHPs indicated a preference for seeking assistance from CHAs rather than other CHPs, primarily because they perceive CHAs as understanding and helpful in clarifying concepts.

Key barriers to peer support among CHPs include vast coverage areas, reluctance of some CHPs to offer help, resistance from others to accept assistance from fellow CHPs, and interpersonal differences among CHPs.

### CHP supplies and equipment

According to CHPs, some CHPs in both counties possessed various supplies and equipment. However, most CHPs had only a few of the required items. Several notable obstacles related to supplies and equipment included delays by CHUs in replenishing CHPs’ supplies, non-functional equipment, a lack of storage containers to ensure the safety of supplies and equipment, insufficient tools for malnutrition detection such as MUAC tapes, a shortage of RUTF for treating acute malnutrition, and limited knowledge among CHPs regarding the utilization of certain supplies and equipment. CHPs stated that lack of necessary supplies and equipment was demotivating.

### CHP incentives

At the time of data collection for this study, Turkana County was issuing a monthly allowance to CHPs. However, Samburu County had not yet enacted its CHS bill and did not offer a stipend to its CHPs. Consequently, participants from Samburu highlighted the absence of monetary incentives as a significant hurdle for CHPs. They reported that while some non-governmental organizations previously provided stipends in Samburu, many of these had ceased doing so, and some were no longer operational.

Certain non-governmental organizations in Turkana offered supplementary stipends in addition to the government-provided stipends. These stipends, along with compensation for training or occasional work at health facilities served as incentives for CHPs. CHPs were demotivated when payments were delayed, as they often were, or when stipends were completely unavailable.

### Experiences with CMAM and family-led MUAC

There were numerous strategies discussed by CHPs and CHAs for enhancing the early detection of acute malnutrition. The most frequently mentioned strategy was the implementation of early screening and CMAM. At the household level, the implementation of family-led MUAC, which encouraged regular and timely screening, was frequently cited as a best practice by CHAs, CHPs, and community members.

Several aspects of acute malnutrition detection and management were functioning effectively in the counties. In Turkana, some CHAs mentioned that they distributed MUAC tapes to families and provided training on their use. CHAs in Samburu and Turkana reported that acute malnutrition was integrated into child welfare clinics, where children diagnosed with acute malnutrition received nutritional supplements and were enrolled in a management and follow-up system. In Samburu, a CHA also highlighted that CHPs facilitated the referral of children diagnosed with acute malnutrition to health facilities for initiation of treatment.

## Discussion

This study investigated the factors influencing acute malnutrition detection and treatment by CHPs in Samburu and Turkana Counties. We based the conceptual framework for our study on literature reviews that developed lists, frameworks, or logic models of factors that influence community health worker performance [21–24]. Previous studies focused on pieces of the frameworks without looking at outcomes (e.g., factors related to community health worker motivation [25–28]) or assessed one to three elements of the framework (e.g., reporting, supervision, incentives, training, equipment) in relation to community health worker performance and health behaviors or outcomes [24]. Our study addressed this gap in the literature by using a framework and examining the linkages between framework constructs.

We found that CHPs’ external motivation (W-NSDM) was associated with acute malnutrition detection, but their internal motivation (W-SDM) was not, and neither part of the motivation scale was associated with acute malnutrition treatment. External motivation is driven by factors such as monetary or non-monetary incentives and feelings of obligation or responsibility, whereas internal motivation is driven by personal goals, autonomy, or ownership of the work [17]. Based on the qualitative data from this study, CHPs felt a strong sense of obligation to their communities to do the work, in part, because they are selected by the community for the job. At the same time, they have specific tasks they are expected to do and do not have much autonomy over those tasks. For example, during the time of this study, CHPs did not have access to RUTF for treating SAM, which may explain the lack of autonomy or ownership (internal motivation) for this part of the work.

CHPs’ self-efficacy, their ability to successfully carry out a specific behavior [29], was related to acute malnutrition detection in both models, but not to acute malnutrition treatment. The relationship between CHPs’ self-efficacy and acute malnutrition detection in this study is in line with the finding by Zamani-Alvijeh et al. (2019), which showed that self-efficacy is crucial for health workers’ success in performing duties [30]. CHPs’ self-efficacy was also associated with internal motivation (W-SDM) in the acute malnutrition detection model, but not internal or external motivation in any of the other models. This aligns with previous workforce research showing that internal motivation is a partial mediator of the relationship between self-efficacy and work performance [31].

Although CHPs talked about the importance of supervision by CHAs and support from their communities in the qualitative data, supervision and social support were not associated with CHPs’ self-efficacy or with CHPs’ motivation in the survey results. This contrasts with findings from Lopez-Ejada et al. (2019) [32] showing that supervision increases community health worker motivation. It was similar to results of an intervention study by Kok et al. (2018) that measured the impact of supportive supervision for community health workers on their motivation and found no consistent quantitative effects, but found that community health workers mentioned the benefits of supervision in the qualitative data [27]. Other studies have shown that supportive feedback boosts health workers’ self-efficacy [30] and that supervisor support in workplaces influences task performance through the mediation of self-efficacy [33].

We found that supervision by CHAs was related to CHPs’ knowledge in all the models and CHPs’ knowledge was also associated with their self-efficacy in all the models. The latter association had the largest coefficient of all the relationships between constructs in the conceptual framework. Surprisingly, training of CHPs was not related to their knowledge in any of the models, although other researchers have found that training increased community health worker knowledge and performance [21,22]. CHPs in these two counties have varying literacy levels, difficulty understanding complex training materials, lack of regular trainings, and long distances to training venues, as described in the qualitative data. It seems likely that what they have learned during training is being retaught or reinforced through supervision and that supervision also makes them feel more confident in their ability to carry out acute malnutrition detection tasks.

We did not find an association of supplies and equipment or stipends with motivation, although other research has shown that stipends can be an important motivator for community health workers [21,34]. The lack of associations in our study are likely related to lack of availability. CHPs described notable obstacles related to supplies and equipment, including delays in replenishing supplies, non-functional equipment, insufficient tools for acute malnutrition detection, a shortage of RUTF, and limited knowledge about using certain supplies and equipment. Our survey and qualitative data also indicated that many CHPs in our study received no stipends and those who did, received them very infrequently (e.g., once per 6 months or less often). Based on the qualitative findings, this was demotivating for many CHPs, whose families depend on their livelihoods.

We did not find associations of the most proximate indicators (CHP self-efficacy and motivation) with acute malnutrition treatment. This may be due to the limited number of CHPs who treated acute malnutrition after detecting it. Another possibility is that the motivation scale we used did not effectively capture CHP motivation. Although the motivation scale is validated [17] and has been used in one other study of CHPs in Kenya [35], it has not been validated in low- and middle-income country (LMIC) contexts. There may be other unobserved factors that we could not account for in the models, including contextual factors that are part of the Kok et al. (2015) model of community health worker performance [22].

### Study strengths and limitations

The study has several strengths. We had a large sample of CHPs and enrolled all CHPs in the selected CHUs. We collected quantitative data to represent the constructs in our conceptual model, which was based on previous community health worker models, and used SEM to understand hypothesized causal pathways. Our quantitative findings were complemented by qualitative data that provided insights into the situation from the perspective of CHPs and their supervisors. This study also had some limitations. Our results showed that contextual factors are important in understanding CHP motivation and self-efficacy, which could limit the generalizability of findings. However, the methods used in this study could be applied in other settings with high GAM prevalence to further understand the causal pathways leading to CHPs’ performance of acute malnutrition detection and treatment tasks in different contexts.

## Conclusion

This study highlights the role of CHP self-efficacy, CHPs’ knowledge, supervision, and external motivation in contributing to CHPs’ performance of acute malnutrition detection. Contextual factors may have limited the effects of social support, supplies and equipment, and stipends on CHPs’ ability to perform acute malnutrition detection. Their performance of acute malnutrition treatment was likely limited by the shortage of RUTF, also a contextual factor highlighted by the qualitative data. Our findings show the relevance of using conceptual models to assess factors influencing CHPs’ performance of key tasks. They also highlight the importance of pairing quantitative and qualitative data to understand CHPs’ motivations and other factors that influence their performance. Future studies could test interventions to improve CHPs’ acute malnutrition detection and treatment and assess how the interventions influence different factors in a conceptual framework as well as further elucidating why some factors are more or less relevant. Research to validate motivation scales for LMICs would also be valuable. Finally, incorporating adaptive learning into action research can help tackle contextual challenges as an integral part of program design.

## Supporting information

S1 Table Sampled community health units (CHUs). (DOCX)

S2 Table Outcome and latent variable descriptions. (DOCX)

S3 Table Percentage of CHPs who have completed training modules. (DOCX)

S4 Table Median number of CHA supervision visits and meetings during the last 3 months reported by CHPs. (DOCX)

S5 Table Percentage of CHPs with knowledge and experience with CMAM and family-led MUAC and median knowledge score. (DOCX)

S6 Table CHP self-efficacy. (DOCX)

S7 Table Percentage of CHPs reporting receiving different types of social and peer support. (DOCX)

S8 Table Percentage of CHPs reporting availability of essential supplies and equipment. (DOCX)

S9 Table Percentage of CHPs receiving stipends and participating in income-generating activities. (DOCX)

S10 Table CHP motivation. (DOCX)

S11 Table Structural equation model showing pathways regression coefficients to acute malnutrition detection through work self-determined motivation (W-SDM). (DOCX)

S12 Table Structural equation model showing pathways regression coefficients to acute malnutrition detection through work non-self-determined motivation (W-NSDM). (DOCX)

S13 Table Structural equation model showing pathways regression coefficients to acute malnutrition treatment through work self-determined motivation (W-SDM). (DOCX)

S14 Table Structural equation model showing pathways regression coefficients to acute malnutrition treatment through work non-self-determined motivation (W-NSDM). (DOCX)
