# Supplementary material for: Factors that influence acute malnutrition detection and treatment by community health promoters in Samburu and Turkana counties, Kenya: A mixed methods study
Source: PLOS Glob Public Health. 2026 Jan 21;6(1):e0005689. doi: 10.1371/journal.pgph.0005689 (PMC12822924; doi:10.1371/journal.pgph.0005689)
Supplement: S1 Table — (DOCX) [file pgph.0005689.s001.docx]

**S1 Table. Sampled community health units (CHUs)**

| **Sub County** | **CHU** |
| --- | --- |
| **Turkana study sites** | |
| **Turkana North** | Kaeris |
|  | Kaaleng |
|  | Kanakurudio |
| **Kibish** | Naita/Kichubi |
|  | Lochilamuya |
|  | Kakelae |
| **Turkana West** | Naduat |
|  | Lokangae |
|  | Aposta |
| **Loima** | Kaitese |
|  | Puch |
|  | Kaapus |
| **Turkana Central** | Kanamkemer |
|  | Kerio |
|  | Ng'itakito |
| **Turkana South** | Katilu |
|  | Kalemng'orok |
|  | Kaputir |
| **Turkana East** | Lokwamosing |
|  | Nakukulas |
|  | Kaaruko |
| **Samburu study sites** | |
| **Samburu Central** | Kisima |
|  | Suguta Marmar |
|  | Loikas-Milimani-Tamiyoi-Ngari-Shabaa |
| **Samburu East** | Sere Olipi |
|  | Lderkesi-Lorubae-Treetop-Kulatanker |
|  | West Gate |
| **Samburu North** | Nachola |
|  | Latakweny |
|  | Bendera-Nalingangor |
