# Supplementary material for: Factors that influence acute malnutrition detection and treatment by community health promoters in Samburu and Turkana counties, Kenya: A mixed methods study
Source: PLOS Glob Public Health. 2026 Jan 21;6(1):e0005689. doi: 10.1371/journal.pgph.0005689 (PMC12822924; doi:10.1371/journal.pgph.0005689)
Supplement: S2 Table — (DOCX) [file pgph.0005689.s002.docx]

## **S2 Table. Outcome and latent variable descriptions**

| **Outcome variables** | **Type** |
| --- | --- |
| **Acute malnutrition detection**  Defined as % of children < 5 years screened who had GAM | Standardized continuous score ranging from 0 to 1. |
| **Acute malnutrition treatment**  Defined as % of GAM cases that received treatment | Standardized continuous score ranging from 0 to 1. |
| **Latent variables** | **Type** |
| **CHP training**  CHPs have a total of 16 possible training modules. CHPs were given a score or one for each training completed or zero if the training was not completed. | Standardized continuous score ranging from 0 to 16. |
| **Supervision by CHA**  Number of supervision activities involving five different items were summed up. Each supervision activity response was a discrete count of the number of supervisions. | Standardized continuous score |
| **CHP knowledge and experience with CMAM and family-led MUAC**  Responses to seven questions about CHPs’ CMAM & family-led MUAC triage and treatment knowledge and experience questions were summed. | Standardized continuous score |
| **CHP self-efficacy**  Responses to eight questions about CHP self-efficacy were summed up. Each question had a scale of five, ranging from a score of one for very low level of confidence to a score of five for very high level of confidence. | Standardized continuous score ranging from 8 to 40. |
| **Social and peer support**  Responses to 11 social support and 4 peer support questions were summed up. The questions had three levels: one for never, two for sometimes, and three for always. | Standardized continuous score ranging from one to 37. |
| **Availability of supplies and equipment**  Yes/no responses to 34 essential supplies and equipment questions were summed up. The responses were scored as zero if absent and one if present. | Standardized continuous score ranging from 0 to 34. |
| **CHP stipends and income generating activities.**  Two yes/no questions, one asking about receipt of a stipend from government/implementing organizations and the other about engagement in income-generating activities, were summed up. | Standardized continuous score ranging from 0 to 2. |
| **CHP motivation**  Two motivation scores were calculated from XX questions in XX format: i) W-SDM (Work self-determined motivation) as the sum of means of Intrinsic motivation (IM), Integrated regulation (INTEG) and Identified regulation (IDEN) and ii) W-NSDM (Work Nonself-Determined Motivation) as sum of means of Introjected regulation (INTRO), External regulation (EXT) and Amotivation (AMO) (9). | Standardized continuous score |
