# Supplementary material for: Factors that influence acute malnutrition detection and treatment by community health promoters in Samburu and Turkana counties, Kenya: A mixed methods study
Source: PLOS Glob Public Health. 2026 Jan 21;6(1):e0005689. doi: 10.1371/journal.pgph.0005689 (PMC12822924; doi:10.1371/journal.pgph.0005689)
Supplement: S3 Table — (DOCX) [file pgph.0005689.s003.docx]

## **S3 Table. Percentage of CHPs who have completed training modules**

| **CHP training modules** | **All participants**  **(N=490)** |
| --- | --- |
|  | N (%) |
| Health & development in the community | 376 (77) |
| Community governance & leadership | 339 (69) |
| Communication advocacy & social mobilization | 344 (70) |
| Best practices for health promotion & disease prevention | 411 (84) |
| Best health care & lifesaving skills | 376 (77) |
| Management & use of community health information | 353 (72) |
| Water, sanitation & hygiene | 436 (89) |
| Community nutrition | 424 (87) |
| Integrated case management | 388 (79) |
| Maternal, newborn & child health | 416 (85) |
| Family planning | 369 (75) |
| TB, Malaria & HIV/AIDS | 328 (67) |
| NCDs & gender-based violence | 254 (52) |
| ICCM-CMAM | 341 (70) |
| Family-led MUAC | 394 (80) |
| COVID-19 | 9 (2) |

^a^Total score (best score) is 16.
