# Supplementary material for: Factors that influence acute malnutrition detection and treatment by community health promoters in Samburu and Turkana counties, Kenya: A mixed methods study
Source: PLOS Glob Public Health. 2026 Jan 21;6(1):e0005689. doi: 10.1371/journal.pgph.0005689 (PMC12822924; doi:10.1371/journal.pgph.0005689)
Supplement: S4 Table — (DOCX) [file pgph.0005689.s004.docx]

## **S4 Table. Median number of CHA supervision visits and meetings during the last 3 months reported by CHPs**

|  | **All participants**  **(N=490)** |
| --- | --- |
|  | Median (IQR) |
| Number of supervisions during household visit in the last 3 months | 2 (0‒3) |
| Number of supervisions during community meetings in the last 3 months | 1 (0‒3) |
| Number of supervisions during outreaches in the last 3 months | 0 (0‒1) |
| Number of group supervision meetings with the supervisor in the last 3 months | 2 (1‒3) |
| Number of group supervision meetings attended in the last 3 months | 3 (2‒3) |
