# Supplementary material for: Factors that influence acute malnutrition detection and treatment by community health promoters in Samburu and Turkana counties, Kenya: A mixed methods study
Source: PLOS Glob Public Health. 2026 Jan 21;6(1):e0005689. doi: 10.1371/journal.pgph.0005689 (PMC12822924; doi:10.1371/journal.pgph.0005689)
Supplement: S5 Table — (DOCX) [file pgph.0005689.s005.docx]

## **S5 Table. Percentage of CHPs with knowledge and experience with CMAM and family-led MUAC and median knowledge score**

| **CMAM & Family Led MUAC Triage and Treatment** | **All participants**  **(N=490)** |
| --- | --- |
|  | N (%) |
| What colors are indicated in a MUAC tape? |  |
| Red | 486 (99) |
| Yellow | 489 (99) |
| Green | 475 (97) |
| What actions or treatment is recommended for a child whose MUAC indicates? |  |
| Color red without complications |  |
| Give RUTF | 315 (64) |
| Refer | 139 (28) |
| Refer to health facility/Hospital | 13 (3) |
| Refer to Nutritional rehabilitation program | 17 (4) |
| Color red with complications |  |
| Refer | 465 (95) |
| Give RUTF/ORS | 2 (0) |
| Color yellow |  |
| Give RUSF | 315 (64) |
| Refer to health facility/ Nutritional rehabilitation program | 98 (20) |
| Provide nutrition counselling | 44 (9) |
| Color green |  |
| Nutrition counselling | 425 (87) |
| How do you determine the dose of RUSF or RUTF to give to a child with acute malnutrition? |  |
| Use weight Dosage Chart to determine RUTF Dosage | 215 (44) |
| Give one Sachet per day for RUSF | 179 (37) |
| When should a child who was diagnosed with acute malnutrition stop receiving RUSF or RUTF? |  |
| When MUAC reads yellow from red in two consecutive Visits | 226 (46) |
| When MUAC reads green from yellow in two consecutive visits, discontinue RUSF and offer nutrition counselling | 371 (76) |
