# Supplementary material for: Factors that influence acute malnutrition detection and treatment by community health promoters in Samburu and Turkana counties, Kenya: A mixed methods study
Source: PLOS Glob Public Health. 2026 Jan 21;6(1):e0005689. doi: 10.1371/journal.pgph.0005689 (PMC12822924; doi:10.1371/journal.pgph.0005689)
Supplement: S6 Table — (DOCX) [file pgph.0005689.s006.docx]

## **S6 Table. CHP self-efficacy**

|  | **All participants**  **(N=490)** |
| --- | --- |
|  | N (%) |
| How confident do you feel about using a MUAC tape? |  |
| Very low level of confidence | 7 (1) |
| Low level of confidence | 15 (3) |
| Medium level of confidence | 77 (16) |
| High level of confidence | 97 (20) |
| Very high level of confidence | 294 (60) |
| How confident do you feel about diagnosing a child as having moderate or severe acute malnutrition? |  |
| Very low level of confidence | 9 (2) |
| Low level of confidence | 28 (6) |
| Medium level of confidence | 89 (19) |
| High level of confidence | 136 (28) |
| Very high level of confidence | 228 (47) |
| How confident do you feel about treating a child with moderate or severe acute malnutrition with RUSF or RUTF? |  |
| Very low level of confidence | 45 (9) |
| Low level of confidence | 86 (18) |
| Medium level of confidence | 124 (25) |
| High level of confidence | 92 (19) |
| Very high level of confidence | 143 (29) |
| How confident do you feel about conducting household visits as part of your role as a CHP? |  |
| Very low level of confidence | 21 (4) |
| Low level of confidence | 51 (10) |
| Medium level of confidence | 139 (28) |
| High level of confidence | 123 (25) |
| Very high level of confidence | 156 (32) |
| How confident do you feel about conducting community activities as part of your role as a CHP? |  |
| Very low level of confidence | 56 (11) |
| Low level of confidence | 104 (21) |
| Medium level of confidence | 140 (28) |
| High level of confidence | 83 (17) |
| Very high level of confidence | 107 (22) |
| How confident do you feel about conducting outreaches as part of your role as a CHP? |  |
| Very low level of confidence | 21 (4) |
| Low level of confidence | 63 (13) |
| Medium level of confidence | 69 (14) |
| High level of confidence | 94 (19) |
| Very high level of confidence | 240 (49) |
| How confident do you feel about filling out referral forms? |  |
| Very low level of confidence | 16 (3) |
| Low level of confidence | 17 (3) |
| Medium level of confidence | 37 (8) |
| High level of confidence | 78 (16) |
| Very high level of confidence | 343 (70) |
| How confident do you feel about filling out data collection tools? |  |
| Very low level of confidence | 18 (4) |
| Low level of confidence | 23 (5) |
| Medium level of confidence | 54 (11) |
| High level of confidence | 88 (18) |
| Very high level of confidence | 309 (63) |
