# Supplementary material for: Factors that influence acute malnutrition detection and treatment by community health promoters in Samburu and Turkana counties, Kenya: A mixed methods study
Source: PLOS Glob Public Health. 2026 Jan 21;6(1):e0005689. doi: 10.1371/journal.pgph.0005689 (PMC12822924; doi:10.1371/journal.pgph.0005689)
Supplement: S7 Table — (DOCX) [file pgph.0005689.s007.docx]

## **S7 Table. Percentage of CHPs reporting receiving different types of social and peer support**

| **Social support** | **All participants**  **(N=490)** |
| --- | --- |
|  | N (%) |
| People in my community told me I was doing my CHV work well |  |
| Never | 7 (1) |
| Sometimes | 140 (29) |
| Always | 343 (70) |
| My supervisor praised my work as a CHV |  |
| Never | 17 (4) |
| Sometimes | 127 (26) |
| Always | 346 (71) |
| My supervisor answered my questions about my CHV activities or reporting |  |
| Never | 48 (10) |
| Sometimes | 117 (24) |
| Always | 325 (66) |
| My supervisor or fellow CHVs helped me do my CHV activities or fill out my reports |  |
| Never | 164 (33) |
| Sometimes | 189 (39) |
| Always | 137 (28) |
| My family helps me with household chores or other tasks so I would have more time for my CHV work |  |
| Never | 77 (16) |
| Sometimes | 120 (24) |
| Always | 293 (60) |
| My family listened to me talk about my CHV work |  |
| Never | 32 (7) |
| Sometimes | 101 (21) |
| Always | 358 (73) |
| My supervisor or fellow CHVs gave me advice about how to do my CHV work |  |
| Never | 24 (5) |
| Sometimes | 191 (39) |
| Always | 275 (56) |
| My supervisor or fellow CHVs show me how to do some of my CHV tasks |  |
| Never | 39 (8) |
| Sometimes | 251 (51) |
| Always | 200 (41) |
| My supervisor or fellow CHVs makes me feel confident even when I make mistakes |  |
| Never | 34 (7) |
| Sometimes | 163 (33) |
| Always | 293 (60) |
| The leaders of my community commend my work as a CHV to my supervisor or in a public meeting |  |
| Never | 49 (10) |
| Sometimes | 179 (37) |
| Always | 262 (53) |
| Staff of the health facility in my CU commended my work as a CHV |  |
| Never | 29 (6) |
| Sometimes | 171 (35) |
| Always | 290 (59) |
| **Peer support** |  |
|  | Median (IQR) |
| In the last 3 months, how often have you participated in CHV peer-to-peer learning activities; median [IQR] | 1 (0–3) |
| In the last 3 months, another CHV helped you with your activities or reporting or have you helped another CHV with activities or reporting? | 246 (50) |
| In the last 3 months, you have visited another high functioning CHU to learn from their experiences? | 97 (20) |
| In the last 3 months, you have participated in online support with other CHVs through SMS or WhatsApp? | 187 (38) |
