# Supplementary material for: Factors that influence acute malnutrition detection and treatment by community health promoters in Samburu and Turkana counties, Kenya: A mixed methods study
Source: PLOS Glob Public Health. 2026 Jan 21;6(1):e0005689. doi: 10.1371/journal.pgph.0005689 (PMC12822924; doi:10.1371/journal.pgph.0005689)
Supplement: S8 Table — (DOCX) [file pgph.0005689.s008.docx]

## **S8 Table. Percentage of CHPs reporting availability of essential supplies and equipment**

| **Essential supplies** | **All participants**  **(N=490)** |
| --- | --- |
|  | N (%) |
| Albendazole | 147 (30) |
| Paracetamol | 108 (22) |
| Tetracycline eye ointment | 107 (22) |
| ORS | 202 (41) |
| Zinc | 190 (39) |
| Iodine solution | 6 (1) |
| MUAC tape | 426 (87) |
| Salter scale | 3 (1) |
| Color coded salter scale | 0 |
| Digital thermometer | 66 (13) |
| ARI timers | 60 (12) |
| First aid box | 10 (2) |
| Rapid diagnostic tests in malaria area | 44 (9) |
| Oral antimalaria | 20 (4) |
| Chlorine/flocculant for turbid water | 55 (11) |
| Chlorine for clear water | 100 (20) |
| Lavibond comparator | 1 (1) |
| ICCM job aids | 71 (14) |
| Family Led MUAC Illustration card | 133 (27) |
| MIYCN Counselling Card | 73 (15) |
| Counting beads | 119 (24) |
| CHV lesson guide | 154 (31) |
| RUTF | 5 (1) |
| RUSF | 4 (1) |
| Combined oral contraceptives | 6 (1) |
| Male condoms | 76 (16) |
| Commodity register | 98 (20) |
| Data collection tools (MoH 514, MoH 513) | 430 (88) |
| Referral forms (MoH 100) | 465 (95) |
| Medical dispensing envelopes | 22 (5) |
| Carrying bag | 124 (23) |
| Stationery (e.g., pens) | 143 (29) |
| Bicycle or access to a bicycle | 51 (10) |
| Mobile phone | 83 (17) |
