# Supplementary material for: Factors that influence acute malnutrition detection and treatment by community health promoters in Samburu and Turkana counties, Kenya: A mixed methods study
Source: PLOS Glob Public Health. 2026 Jan 21;6(1):e0005689. doi: 10.1371/journal.pgph.0005689 (PMC12822924; doi:10.1371/journal.pgph.0005689)
Supplement: S9 Table — (DOCX) [file pgph.0005689.s009.docx]

## **S9 Table. Percentage of CHPs receiving stipends and participating in income-generating activities**

|  | **All participants**  **(N=490)** |
| --- | --- |
|  | N (%) |
| During the past 3 months, received any stipend for work as a CHP | 31 (6) |
| During the past 3 months, participated in any income-generating activities as a CHP | 231 (47) |
