# Supplementary material for: Factors that influence acute malnutrition detection and treatment by community health promoters in Samburu and Turkana counties, Kenya: A mixed methods study
Source: PLOS Glob Public Health. 2026 Jan 21;6(1):e0005689. doi: 10.1371/journal.pgph.0005689 (PMC12822924; doi:10.1371/journal.pgph.0005689)
Supplement: S10 Table — (DOCX) [file pgph.0005689.s010.docx]

## **S10 Table. CHP motivation**

|  |  | **All participants**  **(N=490)** |
| --- | --- | --- |
|  | Reasons for being involved in your work as a CHP |  |
| 1 | Because this is the type of work I chose to do to attain a certain lifestyle (1) |  |
|  | 1 (Does not correspond at all) | 55 (11) |
|  | 2 | 23 (5) |
|  | 3 (Correspond moderately) | 72 (15) |
|  | 4 | 92 (19) |
|  | 5 (Correspond exactly) | 248 (51) |
| 2 | For the income it provides me (2) |  |
|  | 5 (Does not correspond at all) | 221 (45) |
|  | 4 | 71 (14) |
|  | 3 (Correspond moderately) | 89 (18) |
|  | 2 | 37 (8) |
|  | 1 (Correspond exactly) | 72 (15) |
| 3 | I ask myself this question. I don’t seem to be able to manage the important tasks related to this work (3) |  |
|  | 5 (Does not correspond at all) | 137 (28) |
|  | 4 | 108 (22) |
|  | 3 (Correspond moderately) | 151 (31) |
|  | 2 | 49 (10) |
|  | 1 (Correspond exactly) | 45 (9) |
| 4 | Because I derive much pleasure from learning new things (4) |  |
|  | 1 (Does not correspond at all) | 3 (1) |
|  | 2 | 4 (2) |
|  | 3 (Correspond moderately) | 25 (5) |
|  | 4 | 87 (18) |
|  | 5 (Correspond exactly) | 371 (76) |
| 5 | Because it has become a fundamental part of who I am (5) |  |
|  | 1 (Does not correspond at all) | 12 (2) |
|  | 2 | 10 (2) |
|  | 3 (Correspond moderately) | 53 (11) |
|  | 4 | 110 (22) |
|  | 5 (Correspond exactly) | 305 (62) |
| 6 | Because I want to succeed at this job, if not I would be very ashamed of myself (6) |  |
|  | 5 (Does not correspond at all) | 77 (16) |
|  | 4 | 30 (6) |
|  | 3 (Correspond moderately) | 93 (19) |
|  | 2 | 99 (20) |
|  | 1 (Correspond exactly) | 191 (39) |
| 7 | Because I chose this type of work to attain my career goals (7) |  |
|  | 1 (Does not correspond at all) | 40 (8) |
|  | 2 | 33 (7) |
|  | 3 (Correspond moderately) | 87 (18) |
|  | 4 | 118 (24) |
|  | 5 (Correspond exactly) | 212 (43) |
| 8 | For the satisfaction I experience from taking on  interesting challenges (8) |  |
|  | 1 (Does not correspond at all) | 11 (2) |
|  | 2 | 15 (3) |
|  | 3 (Correspond moderately) | 106 (22) |
|  | 4 | 147 (30) |
|  | 5 (Correspond exactly) | 211 (43) |
| 9 | Because it allows me to earn money (9) |  |
|  | 5 (Does not correspond at all) | 228 (47) |
|  | 4 | 69 (14) |
|  | 3 (Correspond moderately) | 90 (18) |
|  | 2 | 31 (6) |
|  | 1 (Correspond exactly) | 72 (15) |
| 10 | Because it is part of the way in which I have chosen to live my life (10) |  |
|  | 1 (Does not correspond at all) | 13 (3) |
|  | 2 | 17 (3) |
|  | 3 (Correspond moderately) | 84 (17) |
|  | 4 | 118 (24) |
|  | 5 (Correspond exactly) | 258 (53) |
| 11 | Because I want to be very good at this work, otherwise I would be very disappointed (11) |  |
|  | 1 (Does not correspond at all) | 48 (10) |
|  | 2 | 39 (8) |
|  | 3 (Correspond moderately) | 94 (19) |
|  | 4 | 133 (27) |
|  | 5 (Correspond exactly) | 176 (36) |
| 12 | I don’t know why, we are provided with unrealistic work conditions (12) |  |
|  | 5 (Does not correspond at all) | 130 (26) |
|  | 4 | 103 (21) |
|  | 3 (Correspond moderately) | 120 (24) |
|  | 2 | 71 (14) |
|  | 1 (Correspond exactly) | 66 (13) |
| 13 | Because I want to be a “winner” in life (13) |  |
|  | 5 (Does not correspond at all) | 62 (13) |
|  | 4 | 34 (7) |
|  | 3 (Correspond moderately) | 86 (18) |
|  | 2 | 110 (22) |
|  | 1 (Correspond exactly) | 198 (40) |
| 14 | Because it is the type of work I have chosen to attain certain important objectives (14) |  |
|  | 1 (Does not correspond at all) | 22 (4) |
|  | 2 | 26 (5) |
|  | 3 (Correspond moderately) | 111 (23) |
|  | 4 | 143 (29) |
|  | 5 (Correspond exactly) | 188 (38) |
| 15 | For the satisfaction I experience when I am successful at doing difficult tasks (15) |  |
|  | 1 (Does not correspond at all) | 11 (2) |
|  | 2 | 23 (5) |
|  | 3 (Correspond moderately) | 99 (20) |
|  | 4 | 151 (31) |
|  | 5 (Correspond exactly) | 206 (42) |
| 16 | Because this type of work provides me with security (16) |  |
|  | 5 (Does not correspond at all) | 56 (11) |
|  | 4 | 36 (7) |
|  | 3 (Correspond moderately) | 77 (16) |
|  | 2 | 118 (24) |
|  | 1 (Correspond exactly) | 203 (41) |
| 17 | I don’t know, too much is expected of us (17) |  |
|  | 5 (Does not correspond at all) | 95 (19) |
|  | 4 | 75 (15) |
|  | 3 (Correspond moderately) | 116 (24) |
|  | 2 | 80 (16) |
|  | 1 (Correspond exactly) | 124 (25) |
| 18 | Because this job is part of my life (18) |  |
|  | 1 (Does not correspond at all) | 16 (3) |
|  | 2 | 15 (3) |
|  | 3 (Correspond moderately) | 69 (14) |
|  | 4 | 142 (29) |
|  | 5 (Correspond exactly) | 248 (51) |
| Motivation scores | |  |
| Work self-determined motivation (W-SDM) | | 12.7 (11.3–13.7) |
| Work Nonself-Determined Motivation (W-NSDM) | | 9.3 (7.7–10.7) |

Footnote: CHP, community health promoter; Intrinsic motivation (IM)=4,8,5; Integrated regulation (INTEG)=5,10,18; Identified regulation (IDEN)=1,7,14; Introjected regulation (INTRO)=6,11,13; External regulation (EXT)=2,9,16; Amotivation (AMO)=3,12,17.
