# Supplementary material for: Factors that influence acute malnutrition detection and treatment by community health promoters in Samburu and Turkana counties, Kenya: A mixed methods study
Source: PLOS Glob Public Health. 2026 Jan 21;6(1):e0005689. doi: 10.1371/journal.pgph.0005689 (PMC12822924; doi:10.1371/journal.pgph.0005689)
Supplement: S11 Table — (DOCX) [file pgph.0005689.s011.docx]

**S11 Table. Structural equation model showing pathways regression coefficients to acute malnutrition detection through work self-determined motivation (W-SDM)**

| **OUTCOME** | **CHP knowledge and experience with CMAM and family-led MUAC** | | **CHP self-efficacy** | | | **Work self-determined motivation (W-SDM)** | | **Increased acute malnutrition detection** | |
| --- | --- | --- | --- | --- | --- | --- | --- | --- | --- |
|  | aRC (95% CI) | *P-value* | aRC (95% CI) | | *P-value* | aRC (95% CI) | *P-value* | aRC (95% CI) | *P-value* |
| CHP training | 0.01 (-0.02, 0.04) | 0.36 | ¶ | | |  | | ¶ | |
| Supervision by CHA | 0.03 (0.007, 0.05) | 0.008 | 0.003 (-0.08, 0.08) | | 0.94 | -0.006 (-0.04, 0.02) | 0.67 |  |  |
| CHP knowledge and experience with CMAM and family-led MUAC | ¶ | | 1.35 (1.02, 1.68) | | <0.001 | ¶ | | ¶ | |
| CHP self-efficacy | ¶ | | ¶ | | | 0.04 (0.01, 0.08) | 0.009 | 0.01 (0.004, 0.02) | 0.003 |
| Social and peer support | ¶ | | 0.08 (-0.003, 0.17) | 0.06 | | 0.03 (-0.001, 0.06) | 0.06 | ¶ | |
| Availability of supplies and equipment | ¶ | | ¶ | | | 0.02 (-0.02, 0.07) | 0.27 | ¶ | |
| CHP stipends and income generating activities | ¶ | | ¶ | | | -0.18 (-0.47, 0.10) | 0.21 | ¶ | |
| Work self-determined motivation (W-SDM) | ¶ | | ¶ | | | ¶ | | 0.01 (-0.005, 0.03) | 0.11 |

*aRC; Adjusted Regression Coefficient, CI; Confidence Intervals, ¶Variable was not included as predictor for the respective outcome; the SEM goodness of fit: chi-square value 2.8, P-value=0.46, Root mean square error of approximation (RMSEA)=0.034 (90%CI 0.028, 0.039) and standardized root mean square (SRMR)=0.058.
