# Supplementary material for: Factors that influence acute malnutrition detection and treatment by community health promoters in Samburu and Turkana counties, Kenya: A mixed methods study
Source: PLOS Glob Public Health. 2026 Jan 21;6(1):e0005689. doi: 10.1371/journal.pgph.0005689 (PMC12822924; doi:10.1371/journal.pgph.0005689)
Supplement: S12 Table — (DOCX) [file pgph.0005689.s012.docx]

## **S12 Table. Structural equation model showing pathways regression coefficients to acute malnutrition detection through work non-self-determined motivation (W-NSDM)**

| **OUTCOME** | **CHP knowledge and experience with CMAM and family-led MUAC** | | **CHP self-efficacy** | | **Work Nonself-Determined Motivation (W-NSDM)** | | **Increased acute malnutrition detection** | |
| --- | --- | --- | --- | --- | --- | --- | --- | --- |
|  | aRC (95% CI) | *P-value* | aRC (95% CI) | *P-value* | aRC (95% CI) | *P-value* | aRC (95% CI) | *P-value* |
| CHP training | 0.01 (-0.02, 0.04) | 0.36 | ¶ | |  | | ¶ | |
| Supervision by CHA | 0.03 (0.007, 0.05) | 0.008 | 0.02 (-0.05, 0.08) | 0.94 | -0.02 (-0.06, 0.02) | 0.41 |  |  |
| CHP knowledge and experience with CMAM and family-led MUAC | ¶ | | 1.35 (1.02, 1.68) | <0.001 | ¶ | | ¶ | |
| CHP self-efficacy | ¶ | | ¶ | | 0.04 (-0.007, 0.08) | 0.10 | 0.01 (0.004, 0.02) | 0.002 |
| Social and peer support | ¶ | | 0.08 (-0.003, 0.17) | 0.06 | -0.02 (-0.07, 0.02) | 0.29 | ¶ | |
| Availability of supplies and equipment | ¶ | | ¶ | | 0.02 (-0.04, 0.08) | 0.53 | ¶ | |
| CHP stipends and income generating activities | ¶ | | ¶ | | -0.17 (-0.57, 0.24) | 0.43 | ¶ | |
| Work Nonself-Determined Motivation (W-NSDM) | ¶ | | ¶ | | ¶ | | 0.02 (0.007, 0.03) | 0.003 |

*aRC; Adjusted Regression Coefficient, CI; Confidence Intervals, ¶Variable was not included as predictor for the respective outcome; the SEM goodness of fit: chi-square value 1.9, P-value=0.24, Root mean square error of approximation (RMSEA)=0.043 (90%CI 0.038, 0.049) and standardized root mean square (SRMR)=0.059.
