# Supplementary material for: Factors that influence acute malnutrition detection and treatment by community health promoters in Samburu and Turkana counties, Kenya: A mixed methods study
Source: PLOS Glob Public Health. 2026 Jan 21;6(1):e0005689. doi: 10.1371/journal.pgph.0005689 (PMC12822924; doi:10.1371/journal.pgph.0005689)
Supplement: S14 Table — (DOCX) [file pgph.0005689.s014.docx]

## **S14 Table. Structural equation model showing pathways regression coefficients to acute malnutrition treatment through work nonself-determined motivation (W-NSDM)**

| **OUTCOME** | **CHP knowledge and experience with CMAM and family-led MUAC** | | **CHP self-efficacy** | | **Work Nonself-Determined Motivation (W-NSDM)** | | **Increased acute malnutrition treatment** | |
| --- | --- | --- | --- | --- | --- | --- | --- | --- |
|  | aRC (95% CI) | *P-value* | aRC (95% CI) | *P-value* | aRC (95% CI) | *P-value* | aRC (95% CI) | *P-value* |
| CHP training | 0.01 (-0.02, 0.04) | 0.60 | ¶ | |  | | ¶ | |
| Supervision by CHA | 0.03 (0.01, 0.05) | 0.004 | 0.02 (-0.07, 0.11) | 0.65 | -0.02 (-0.06, 0.03) | 0.41 |  |  |
| CHP knowledge and experience with CMAM and family-led MUAC | ¶ | | 1.41 (1.02, 1.80) | <0.001 | ¶ | | ¶ | |
| CHP self-efficacy | ¶ | | ¶ | | 0.02 (-0.03, 0.07) | 0.41 | 0.004 (-0.01, 0.02) | 0.61 |
| Social and peer support | ¶ | | 0.05 (-0.05, 0.15) | 0.30 | -0.04 (-0.09, 0.008) | 0.10 | ¶ | |
| Availability of supplies and equipment | ¶ | | ¶ | | 0.03 (-0.04, 0.09) | 0.36 | ¶ | |
| CHP stipends and income generating activities | ¶ | | ¶ | | -0.16 (-0.59, 0.28) | 0.47 | ¶ | |
| Work Nonself-Determined Motivation (W-NSDM) | ¶ | | ¶ | | ¶ | | -0.003 (-0.03, 0.03) | 0.85 |

*aRC; Adjusted Regression Coefficient, CI; Confidence Intervals, ¶Variable was not included as predictor for the respective outcome
